# Defining Tumor-Induced Iliacus Syndrome: A Case Report on Radiotherapy and Gait Recovery

**DOI:** 10.7759/cureus.85894

**Published:** 2025-06-13

**Authors:** Yojiro Ishikawa, Satoshi Teramura, Kengo Ito, Takayuki Yamada

**Affiliations:** 1 Division of Radiology, Tohoku Medical and Pharmaceutical University, Sendai, JPN

**Keywords:** malignant hip flexion failure, malignat psoas syndrome, palliative radiation therapy, tumor-induced iliacus dysfunction, tumor-induced muscle dysfunction

## Abstract

Tumor invasion of the iliacus muscle is rare, and its clinical implications remain unclear. While malignant psoas syndrome (MPS) is characterized by severe pain and hip flexion contracture due to psoas muscle involvement, isolated iliacus muscle infiltration presents differently. In this report, we propose tumor-induced iliacus syndrome as a distinct clinical entity characterized by gait initiation impairment and slowed walking speed rather than fixed contracture or complete hip flexion failure. A woman in her sixties was diagnosed with stage IVB ovarian endometrioid carcinoma after routine lung cancer screening revealed abnormal diaphragmatic shadows. Imaging studies identified peritoneal nodules, a left ovarian mass, and a lytic bone lesion in the right iliac bone. A biopsy confirmed ovarian endometrioid carcinoma. She underwent chemotherapy with carboplatin and paclitaxel but developed sudden gait impairment without neurological abnormalities on brain and spinal imaging. Despite being able to stand and walk slowly with assistance, she experienced delays initiating each step, requiring a conscious effort to move her leg forward. Follow-up imaging showed iliacus muscle thickening and infiltration adjacent to the enlarging iliac bone lesion. Given her progressive gait impairment and worsening pain, chemotherapy was discontinued, and palliative radiotherapy was initiated. She received a total dose of 40 Gy in 15 fractions using 10 MV and 6 MV X-ray beams, targeting the iliac bone lesion and iliacus muscle involvement. Initially, she required a wheelchair due to difficulty initiating movement. After radiotherapy, her ambulatory function improved, allowing her to walk independently with mild residual gait disturbances. This case introduces tumor-induced iliacus syndrome as a newly recognized clinical entity distinct from MPS. Unlike MPS, which presents with severe pain and hip flexion contracture, iliacus muscle invasion predominantly affects gait initiation and walking speed. Recognizing this syndrome may improve diagnostic accuracy and optimize treatment strategies. Further research and case accumulation are needed to define its clinical significance and establish appropriate management approaches.

## Introduction

The iliacus muscle, which, together with the psoas major, comprises the iliopsoas muscle group, plays a crucial role in maintaining normal gait by generating the force needed to propel the lower limb forward during ambulation [1-2]. Malignant conditions affecting the iliopsoas muscle group have traditionally been associated with involvement of the psoas major, as seen in malignant psoas syndrome (MPS) and hip flexion failure syndrome, where patients typically experience severe pain, hip flexion fixation, and impaired hip mobility [3-6]. However, isolated tumor invasion of the iliacus muscle is rare, and few cases have been described in detail. Due to its anatomical characteristics, the iliacus muscle is crucial for initiating the forward swing of the lower limb and for maintaining walking speed. Localized tumor involvement may lead to more subtle motor deficits than those observed in conventional MPS or hip flexion dysfunction syndrome, such as difficulty with gait initiation and reduced walking speed [7-10]. Therefore, this report focuses on the unique clinical presentation resulting from isolated malignant tumor invasion of the iliacus muscle and proposes the term “tumor-induced iliacus syndrome” to clearly distinguish it from conventional syndromes. This new definition is expected to clarify diagnostic criteria, reduce the risk of misdiagnosis, and facilitate the development of appropriate treatment strategies, ultimately standardizing future research and improving clinical management.

## Case presentation

**Figure 1:**
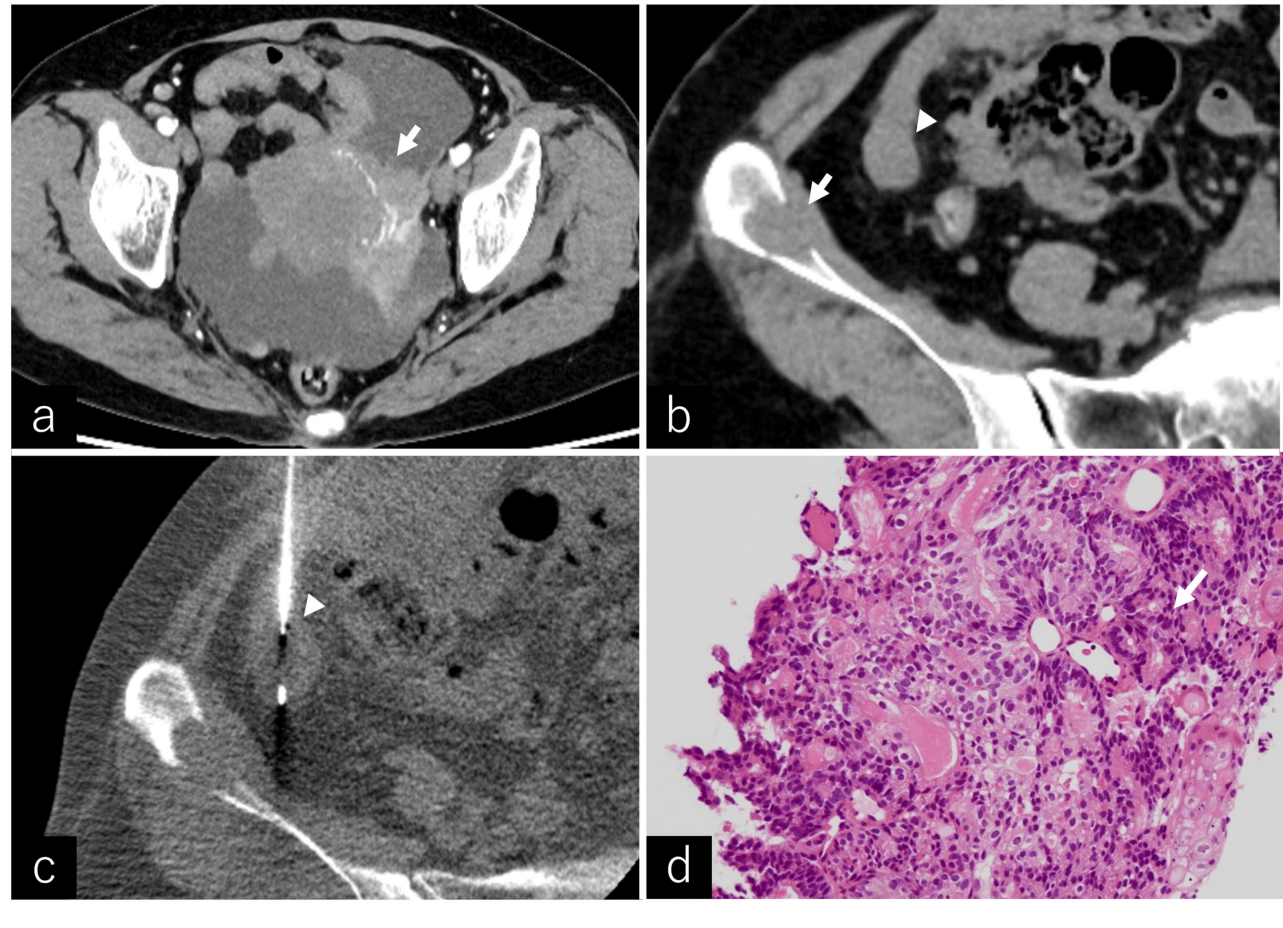
CT and pathology images CT: computed tomography The CT images and pathological findings of ovarian cancer are presented. (a) shows the primary ovarian tumor (arrow) with ascites in the surrounding area. (b) demonstrates an iliac metastatic lesion (arrow) and peritoneal dissemination (arrowhead). (c) depicts a CT-guided biopsy of the peritoneal dissemination lesion. (d) presents the pathological findings of ovarian cancer using hematoxylin and eosin staining at ×10 objective magnification, revealing atypical cells with hyperchromatic and enlarged nuclei proliferating and forming irregular glandular structures

A woman in her sixties, a former smoker with a history of 20 cigarettes per day for 40 years, underwent lung cancer screening, which revealed an abnormal diaphragmatic shadow. Further evaluation with computed tomography (CT) and magnetic resonance imaging (MRI) demonstrated a left ovarian mass measuring 63 × 60 × 51 mm, peritoneal dissemination, right diaphragmatic infiltration, retroperitoneal and pericardiophrenic lymphadenopathy, ascites, and a lytic bone lesion in the right iliac bone measuring 18 × 12 × 15 mm (Figures 1a-1b). A biopsy of the peritoneal nodules confirmed a diagnosis of ovarian endometrioid carcinoma, classified as stage IVB (T3N1M1) according to the International Federation of Gynecology and Obstetrics (FIGO) 2014 staging system (Figures 1c-1d).

She initially received two cycles of carboplatin and paclitaxel. During treatment, bone metastases progressed, accompanied by new gait disturbance and right hip pain. Gait impairment developed suddenly, but neurological exams and brain/spinal imaging showed no central nervous system (CNS) involvement. Her mobility declined, requiring a wheelchair, though she could still stand with help and walk slowly with difficulty. She reported delayed step initiation, needing to consciously plan each movement (Videos 1, 2).

**Video 1:** Gait at initial presentation This video captures the patient's gait at the time of the initial presentation. Despite being instructed to walk, the patient was unable to take the first step immediately and had difficulty initiating movement. Even after starting to walk, the gait remained slow and cautious. Although the patient did not complain of pain, the walking pattern resembled that of an antalgic gait

**Video 2:** Hip joint movement at initial presentation This video demonstrates the patient's hip joint movement at the time of the initial presentation. Although gait was slow, lower limb movement in the seated position was smooth. No pain was observed during hip flexion

**Figure 2:**
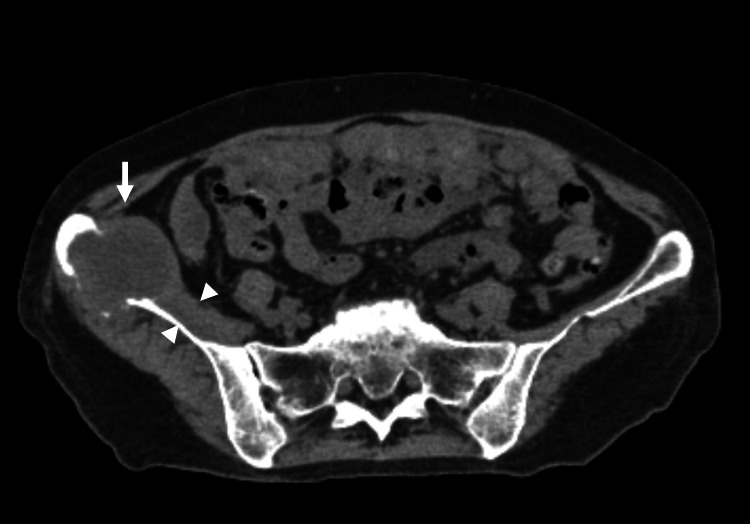
CT imaging at initial radiation oncology consultation CT: computed tomography The CT scan from the initial radiation oncology consultation reveals an enlarging mass in the right iliac bone with osteolytic bone metastasis (arrow). Additionally, the right psoas muscle appears hypertrophied compared to the left (arrowheads)

Follow-up imaging revealed progression of the right iliac bone lesion, which increased to 40 × 34 × 48 mm, with iliacus muscle thickening and surrounding low-attenuation areas, suggesting tumor infiltration (Figure 2). Given the deterioration in mobility and worsening pain, chemotherapy was discontinued, and she was referred for palliative radiotherapy.

**Figure 3:**
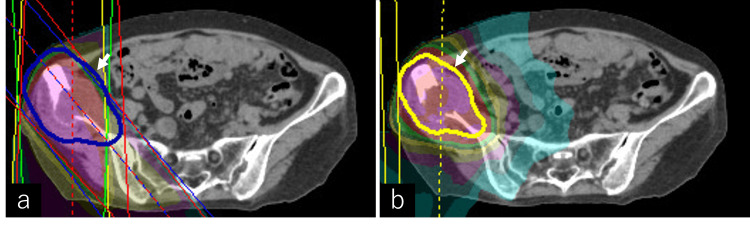
Dose distribution of radiation therapy This figure illustrates the dose distribution of radiation therapy. (a) shows the dose distribution for localized irradiation at 20 Gy/five fractions using 3D conformal radiotherapy (3DCRT). To achieve prompt symptom relief, five initial fractions of 3DCRT were administered as a priority. The arrow indicates the planned target volume (PTV). (b) represents the treatment plan using volumetric modulated arc therapy (VMAT), delivering 20 Gy/10 fractions, with the arrow also marking the PTV

A radiotherapy plan was developed targeting the iliac bone metastasis and iliacus muscle involvement. The gross tumor volume (GTV) included the iliac bone lesion and iliacus muscle involvement, while the clinical target volume (CTV) encompassed the GTV with a 0.5 cm margin. A planning target volume (PTV) was defined by adding another 0.5 cm margin to the CTV. The treatment was initially planned to deliver 39 Gy in 13 fractions (3 Gy per fraction), aiming for effective palliation with a high biological effective dose (BED). However, the schedule was modified due to an unavoidable one-week interruption during the New Year holidays. The patient first received 20 Gy in five fractions with 10 MV X-rays before the break, followed by 20 Gy in 10 fractions with 6 MV X-rays after the break, while maintaining an equivalent BED to the original plan (approximately 52 Gy in both regimens) (Figures 3a-3b).

After radiotherapy, her symptoms gradually improved. By the final evaluation, she had regained ambulatory function, walking independently with only mild gait disturbances (Video 3). She reported feeling more confident in her movements and was able to perform daily activities, such as walking to the restroom, without assistance.

**Video 3:** Gait after radiation therapy This video captures the patient's gait one week after receiving 20 Gy/five fractions of radiation therapy. The patient's walking became noticeably smoother compared to the initial presentation

## Discussion

The iliacus muscle plays a critical role in generating the force necessary for leg swing during gait. As gait speed decreases, muscle activity patterns shift [8], and intramuscular energy flow is also altered [9]. Previous studies have shown that gait speed is closely linked to muscle function, with local dysfunction, such as that caused by tumor invasion, contributing to impaired gait initiation and reduced walking speed [10,11]. The present case illustrates how isolated invasion of the iliacus muscle can disrupt gait, highlighting the clinical significance of tumor involvement in this muscle.

Traditionally, MPS is associated with severe pain and fixed hip flexion due to tumor infiltration of the entire iliopsoas muscle. Malignant hip flexion failure syndrome, in contrast, primarily presents as impaired hip flexion [3-6]. This case, involving isolated iliacus muscle invasion, displayed a distinct clinical picture characterized by difficulty initiating gait and overall gait slowing, differing from both conventional syndromes. Recognizing this presentation as a separate clinical entity underscores the importance of identifying iliacus muscle involvement as a specific cause of gait impairment.

Both MPS and malignant hip flexion failure syndrome are difficult to diagnose due to nonspecific symptoms, such as pain and movement disorders, which often mimic orthopedic or neurological conditions [3,4]. When the iliacus muscle is involved, the risk of misdiagnosis increases, particularly in the absence of pathological confirmation symptoms that may resemble those of nontraumatic lesser trochanteric fractures or other musculoskeletal abnormalities [5,6,12-16]. This case emphasizes the need to recognize tumor-induced iliacus dysfunction as a distinct clinical condition to enhance diagnostic accuracy. Establishing diagnostic criteria based on hallmark symptoms such as gait initiation difficulty and reduced walking speed improves clinical recognition, guides appropriate rehabilitation strategies, and supports standardized research. However, this proposal is based on a single case, and symptom variability may complicate efforts to define consistent diagnostic standards. Differences in assessment methods and reliance on self-reported symptoms further contribute to diagnostic uncertainty. Additional case reports and multicenter studies are necessary to validate the clinical relevance and applicability of this proposed entity.

## Conclusions

In this case, the presence of a malignant tumor resulted in an atypical clinical picture characterized by difficulty initiating walking and a general reduction in walking speed. This presentation differs from previously reported cases of MPS, typically associated with severe pain and hip flexion fixation, and malignant hip flexion failure syndrome, which is marked by hip flexion difficulty. The findings of this study lay the foundation for a novel definition of neoplastic changes in the iliopsoas muscle, termed “tumor-induced iliacus syndrome.” This new definition is anticipated to contribute to more precise diagnostic criteria, a reduction in misdiagnoses, and the development of appropriate treatment strategies. 
